# Supplementary material for: Causal interplay between lactose intolerance and gut microbiota: a combined bidirectional Mendelian randomization and in vivo validation study
Source: Front Nutr. 2026 Jun 1;13:1803337. doi: 10.3389/fnut.2026.1803337 (PMC13265576; doi:10.3389/fnut.2026.1803337)
Supplement: Supplementary file 1 [file Data_Sheet_1.zip › supplementary materials/Forward/leave one out cross validation/ebi-a-GCST90027520.finngen_R12_E4_LACTONAS.pdf]

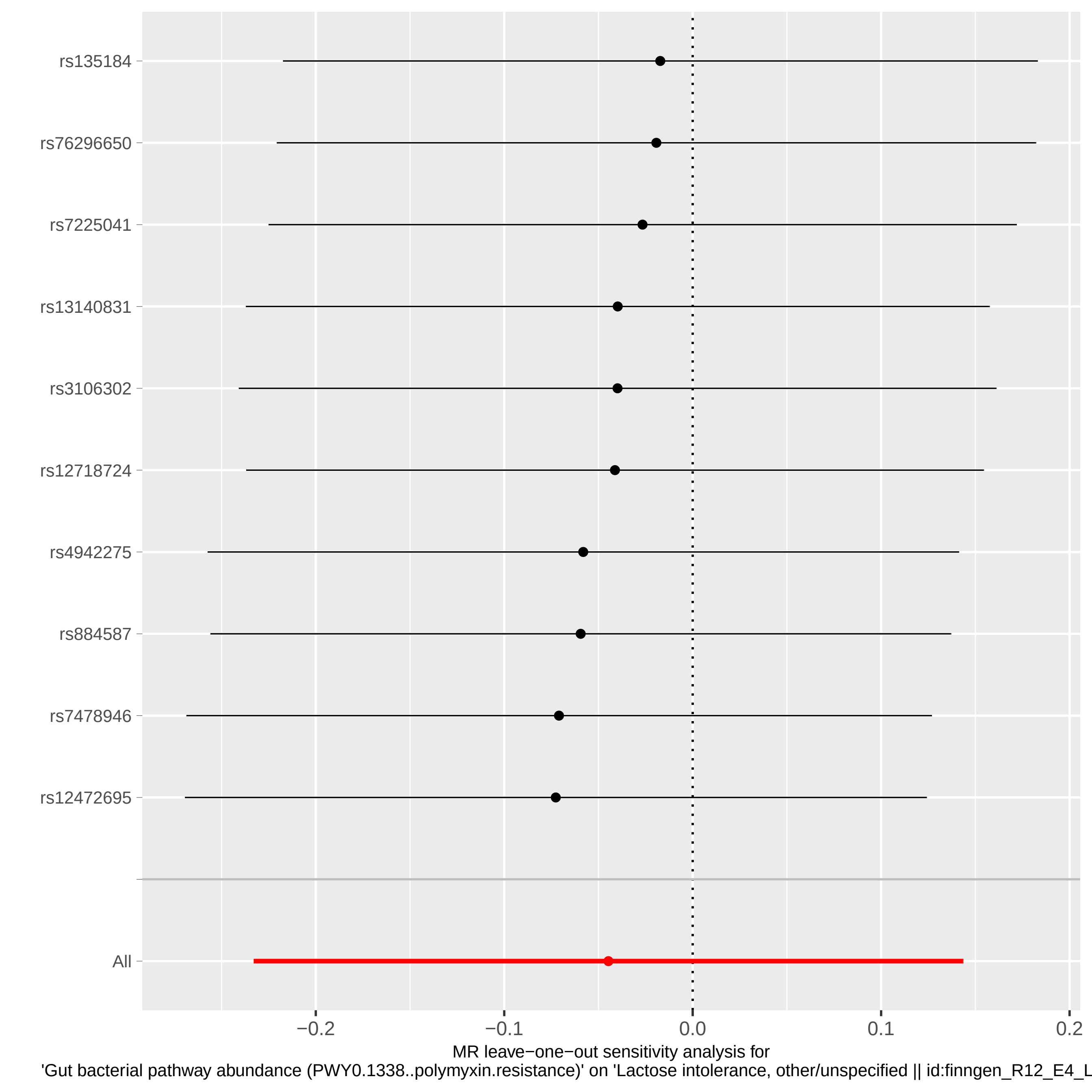

rs135184
rs76296650
rs7225041
rs13140831
rs3106302
rs12718724
rs4942275
rs884587
rs7478946
rs12472695
All
−0.2
−0.1
0.0
0.1
0.2
MR leave−one−out sensitivity analysis for
'Gut bacterial pathway abundance (PWY0.1338..polymyxin.resistance)' on 'Lactose intolerance, other/unspecified || id:finngen_R12_E4_L
